# Supplementary material for: Historic methicillin-resistant Staphylococcus aureus: expanding current knowledge using molecular epidemiological characterization of a Swiss legacy collection
Source: Genome Med. 2024 Feb 5;16:23. doi: 10.1186/s13073-024-01292-w (PMC10840241; doi:10.1186/s13073-024-01292-w)
Supplement: Supplementary file 1 — Additional file 1. This file contains all supplementary figures and tables described in the manuscript. A table of ST distribution followed by four cgMLST multiple spanning trees and nine maximum likelihood phylogenetic trees. [file 13073_2024_1292_MOESM1_ESM.pdf]

A. Table of MLST sequence type distribution in different datasets

ST	Total frequency (n=1761)	Frequency in CCOS (n=451)	Frequency in Public Repositories (n=103)	Frequency in University Hospital Basel (n=1207)
247	408 (23.2%)	376 (83.4%)	29 (28.2%)	3 (0.2%)
22	214 (12.2%)		11 (10.7%)	203 (16.8%)
5	153 (8.7%)	1 (0.2%)	7 (6.8%)	145 (12%)
8	145 (8.2%)		4 (3.9%)	141 (11.7%)
398	93 (5.3%)			93 (7.7%)
?	73 (4.1%)	1 (0.2%)		72 (6%)
1	73 (4.1%)			73 (6%)
6	72 (4.1%)			72 (6%)
30	56 (3.2%)			56 (4.6%)
45	51 (2.9%)			51 (4.2%)
225	41 (2.3%)			41 (3.4%)
239	39 (2.2%)	15 (3.3%)	15 (14.6%)	9 (0.7%)
250	37 (2.1%)	7 (1.6%)	30 (29.1%)	
7844	25 (1.4%)	25 (5.5%)		
2626	23 (1.3%)			23 (1.9%)
1472	20 (1.1%)			20 (1.7%)
254	18 (1%)	18 (4%)		
72	18 (1%)			18 (1.5%)
80	18 (1%)			18 (1.5%)
88	14 (0.8%)			14 (1.2%)
1232	13 (0.7%)			13 (1.1%)
59	13 (0.7%)			13 (1.1%)
97	12 (0.7%)			12 (1%)
152	10 (0.6%)			10 (0.8%)
4080	7 (0.4%)			7 (0.6%)
105	6 (0.3%)			6 (0.5%)
672	6 (0.3%)			6 (0.5%)
1529	5 (0.3%)		5 (4.9%)	
1637	5 (0.3%)			5 (0.4%)
94	5 (0.3%)			5 (0.4%)
130	4 (0.2%)			4 (0.3%)
149	4 (0.2%)			4 (0.3%)
188	4 (0.2%)			4 (0.3%)
49	4 (0.2%)			4 (0.3%)
125	3 (0.2%)			3 (0.2%)
1413	3 (0.2%)			3 (0.2%)
2802	3 (0.2%)			3 (0.2%)
368	3 (0.2%)			3 (0.2%)
737	3 (0.2%)			3 (0.2%)
772	3 (0.2%)			3 (0.2%)
834	3 (0.2%)			3 (0.2%)
113	2 (0.1%)			2 (0.2%)
121	2 (0.1%)			2 (0.2%)
241	2 (0.1%)			2 (0.2%)
4994	2 (0.1%)			2 (0.2%)
7	2 (0.1%)			2 (0.2%)
7119	2 (0.1%)			2 (0.2%)
7638	2 (0.1%)	2 (0.4%)		
7839	2 (0.1%)	2 (0.4%)		
789	2 (0.1%)			2 (0.2%)
923	2 (0.1%)			2 (0.2%)
111	1 (0.1%)			1 (0.1%)
1153	1 (0.1%)			1 (0.1%)
1245	1 (0.1%)			1 (0.1%)
1327	1 (0.1%)			1 (0.1%)
1456	1 (0.1%)			1 (0.1%)
146	1 (0.1%)			1 (0.1%)
1649	1 (0.1%)			1 (0.1%)
207	1 (0.1%)			1 (0.1%)
2249	1 (0.1%)		1 (1%)	
228	1 (0.1%)			1 (0.1%)
2371	1 (0.1%)			1 (0.1%)
2625	1 (0.1%)			1 (0.1%)
3108	1 (0.1%)			1 (0.1%)
3124	1 (0.1%)			1 (0.1%)
337	1 (0.1%)		1 (1%)	
338	1 (0.1%)			1 (0.1%)
36	1 (0.1%)			1 (0.1%)
39	1 (0.1%)			1 (0.1%)
3923	1 (0.1%)			1 (0.1%)
4006	1 (0.1%)			1 (0.1%)
5004	1 (0.1%)			1 (0.1%)
5184	1 (0.1%)			1 (0.1%)
630	1 (0.1%)			1 (0.1%)
6864	1 (0.1%)			1 (0.1%)
718	1 (0.1%)			1 (0.1%)
7837	1 (0.1%)	1 (0.2%)		
7842	1 (0.1%)	1 (0.2%)		
7843	1 (0.1%)	1 (0.2%)		
87	1 (0.1%)			1 (0.1%)
9	1 (0.1%)	1 (0.2%)		
939	1 (0.1%)			1 (0.1%)

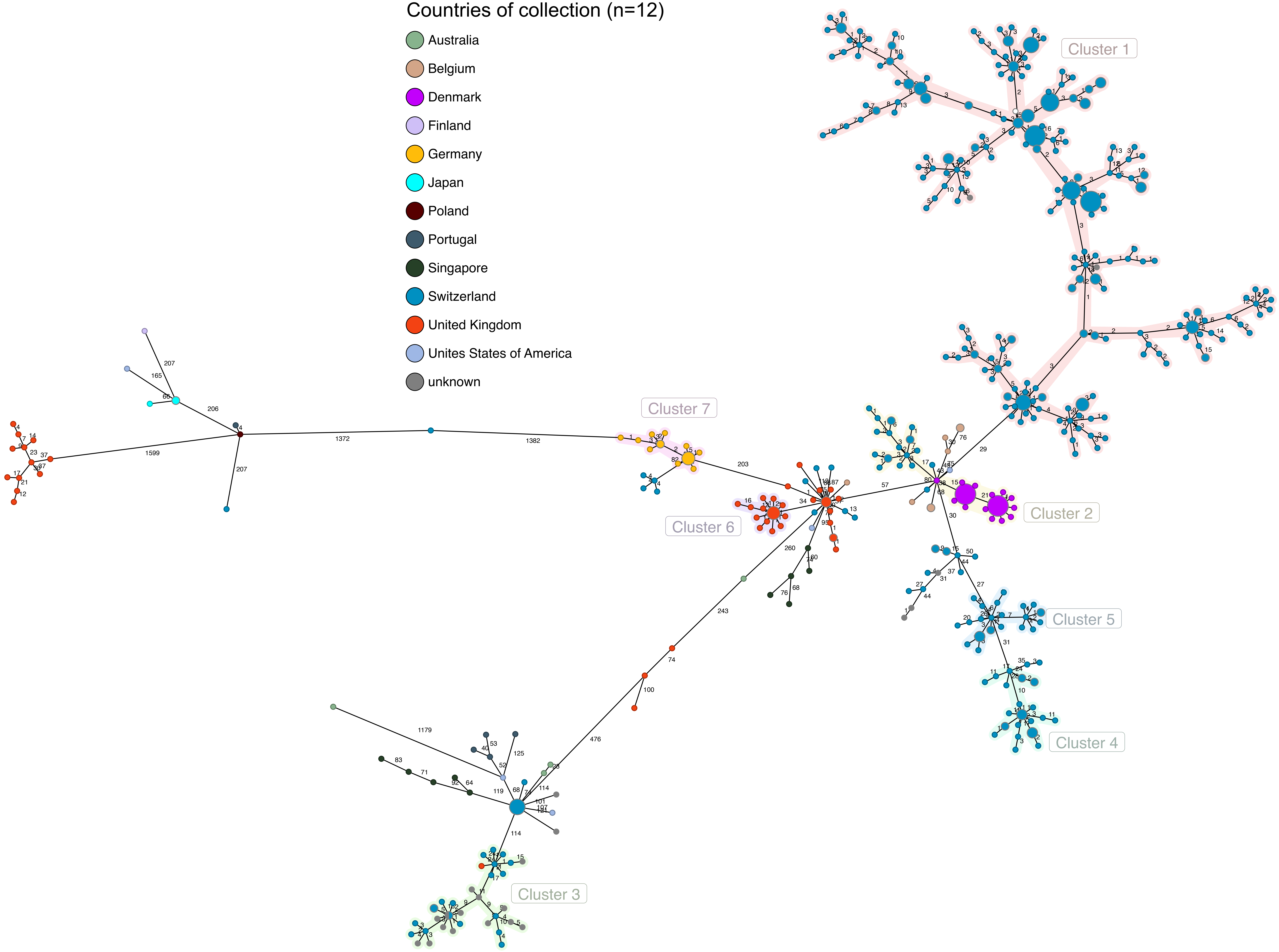

B. cgMLST MST of CCoS and public repository genomes of MRSA (1960-1992, n = 554), nodes colored by collection country. Clusters are shown with a maximal cluster distance of 24 allele differences and a minimal cluster size of 15.

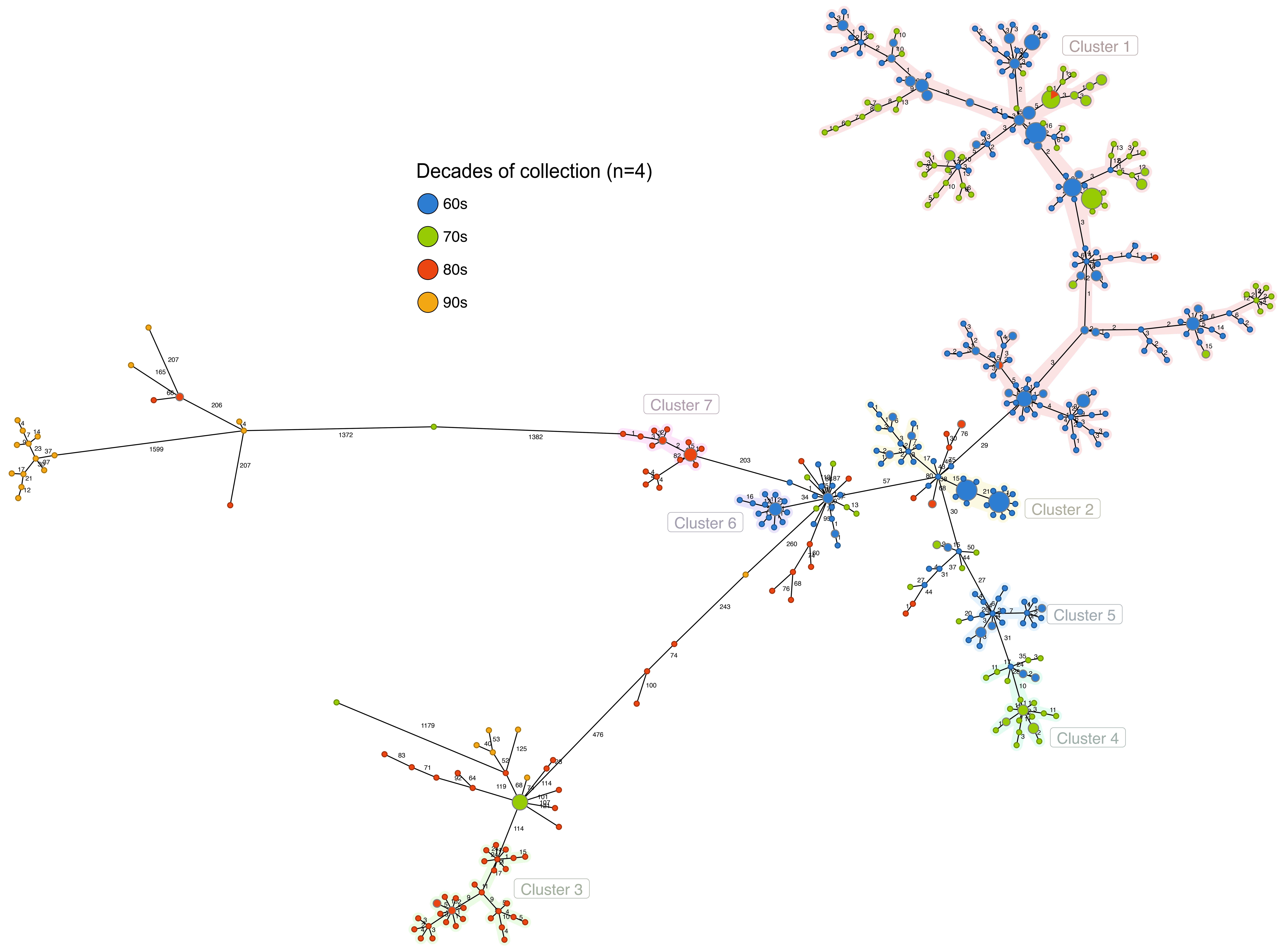

C. cgMLST MST of CCoS and public repository genomes of MRSA (1960-1992, n = 554), nodes colored by sequence type and CCs are shaded. Clusters are shown with a maximal cluster distance of 24 allele differences and a minimal cluster size of 15.

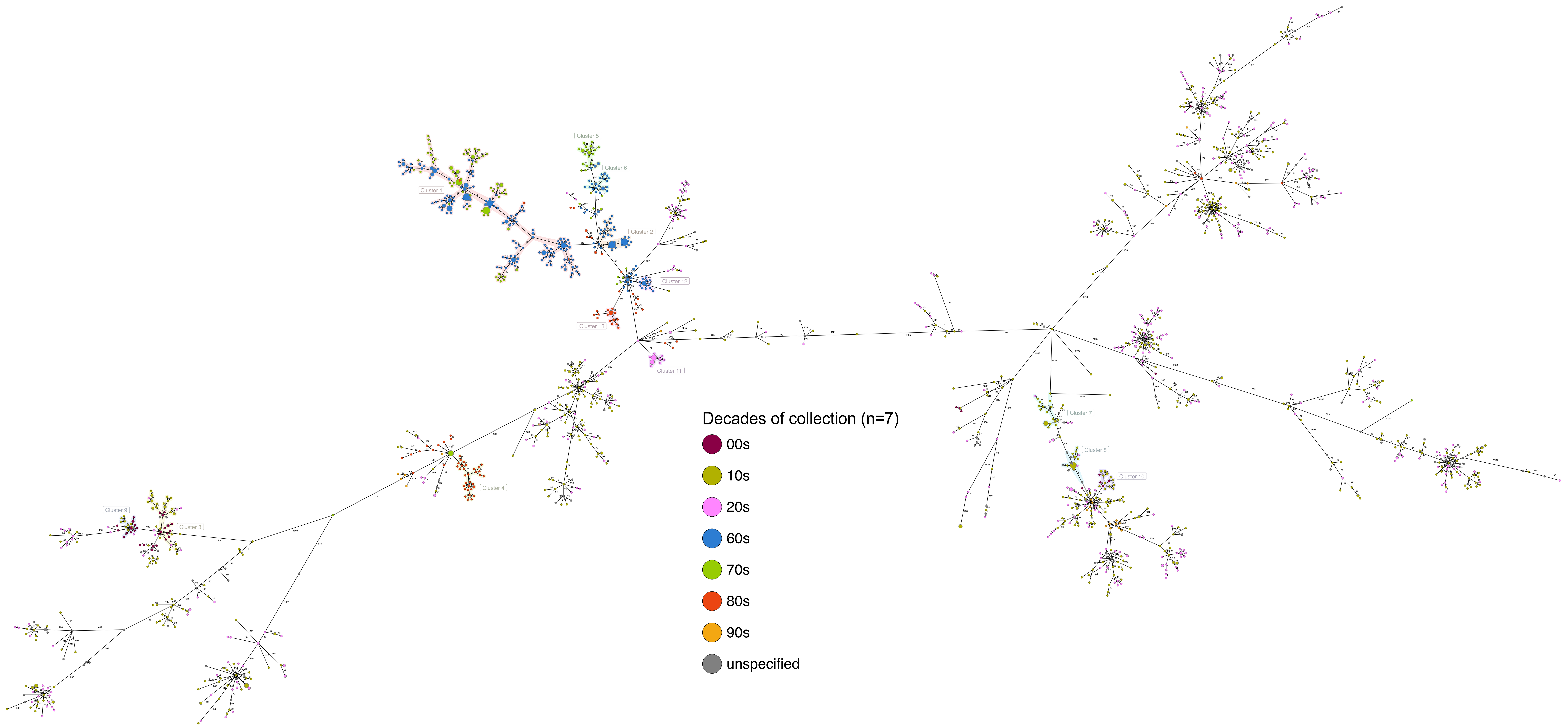

D. cgMLST MST of CCoS, public repository and University Hospital of Basel genomes of MRSA (1960-2022, n = 1761), nodes colored by collection decade. Clusters are shown with a maximal cluster distance of 24 allele differences and a minimal cluster size of 15.

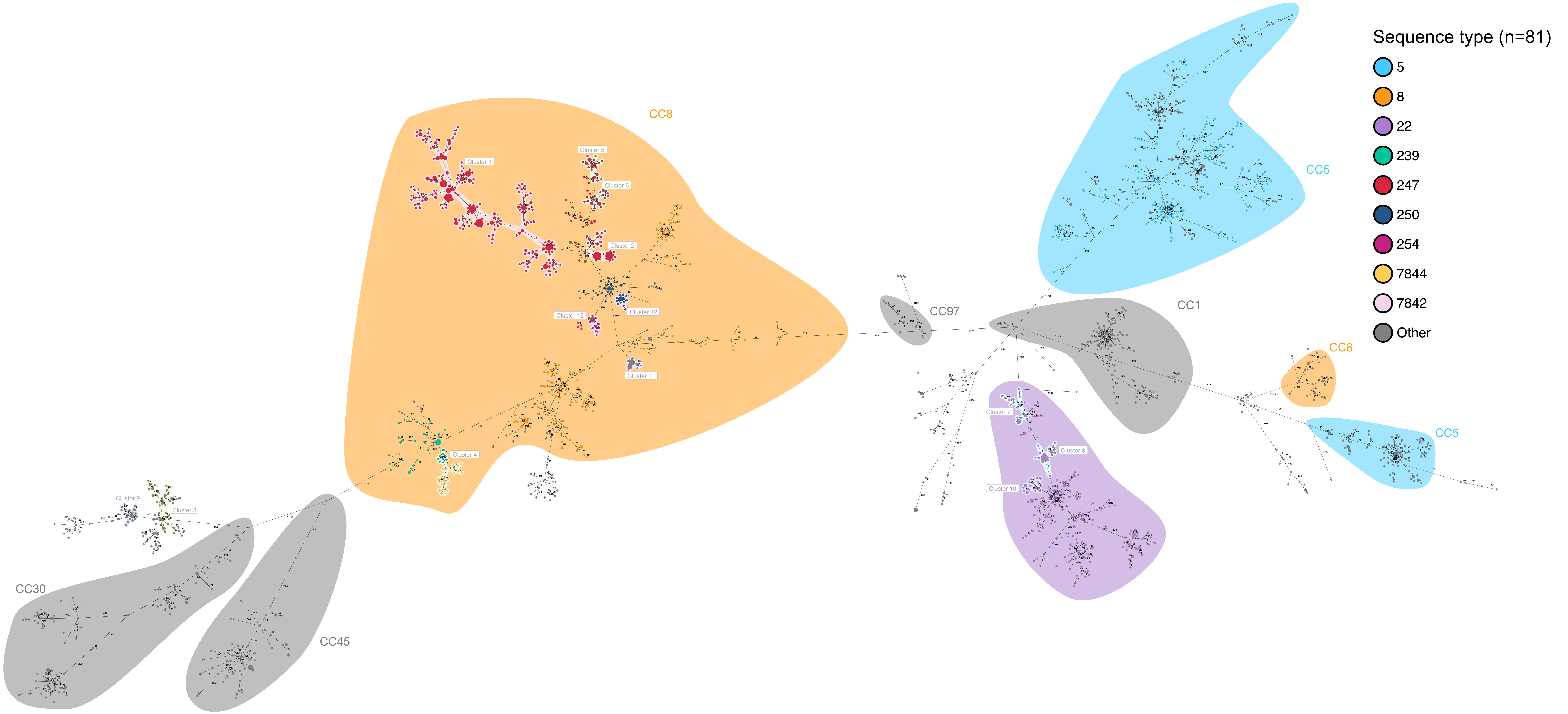

E. cgMLST MST of CCoS, public repository and University Hospital of Basel genomes of MRSA (1960-2022, n = 1761), nodes colored by collection country. Clusters are shown with a maximal cluster distance of 24 allele differences and a minimal cluster size of 15. Clonal complexes are shaded in.

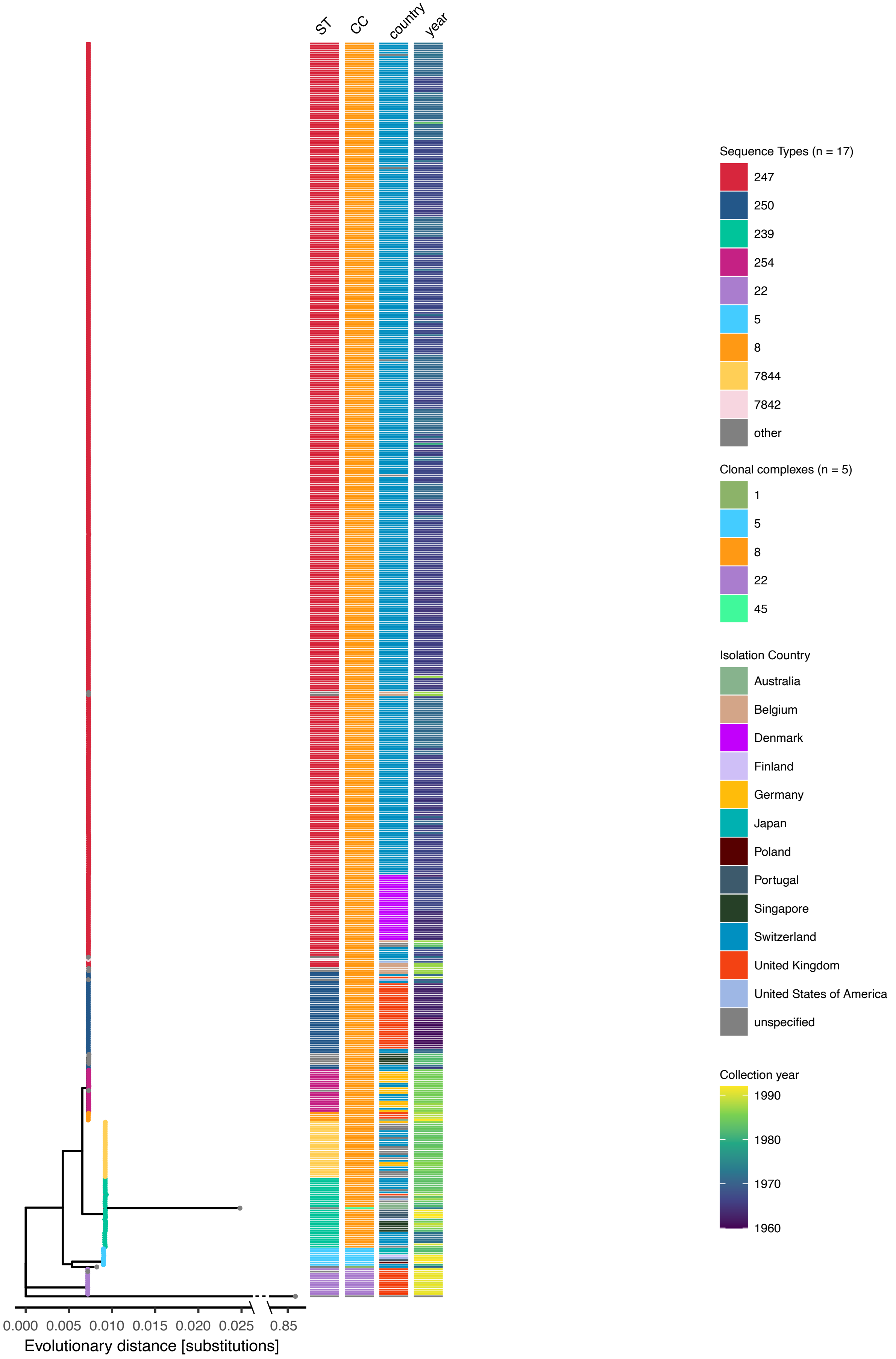

F. MLST sequence type, clonal complex, collection country and collection year mapped to a maximum likelihood core genome SNP tree of ancient Swiss and international MRSA (1960 -1992, n = 554). Leaves colored by sequence type. Outgroup (S. epidermis) line shortened through X-axis break for visualization purposes.

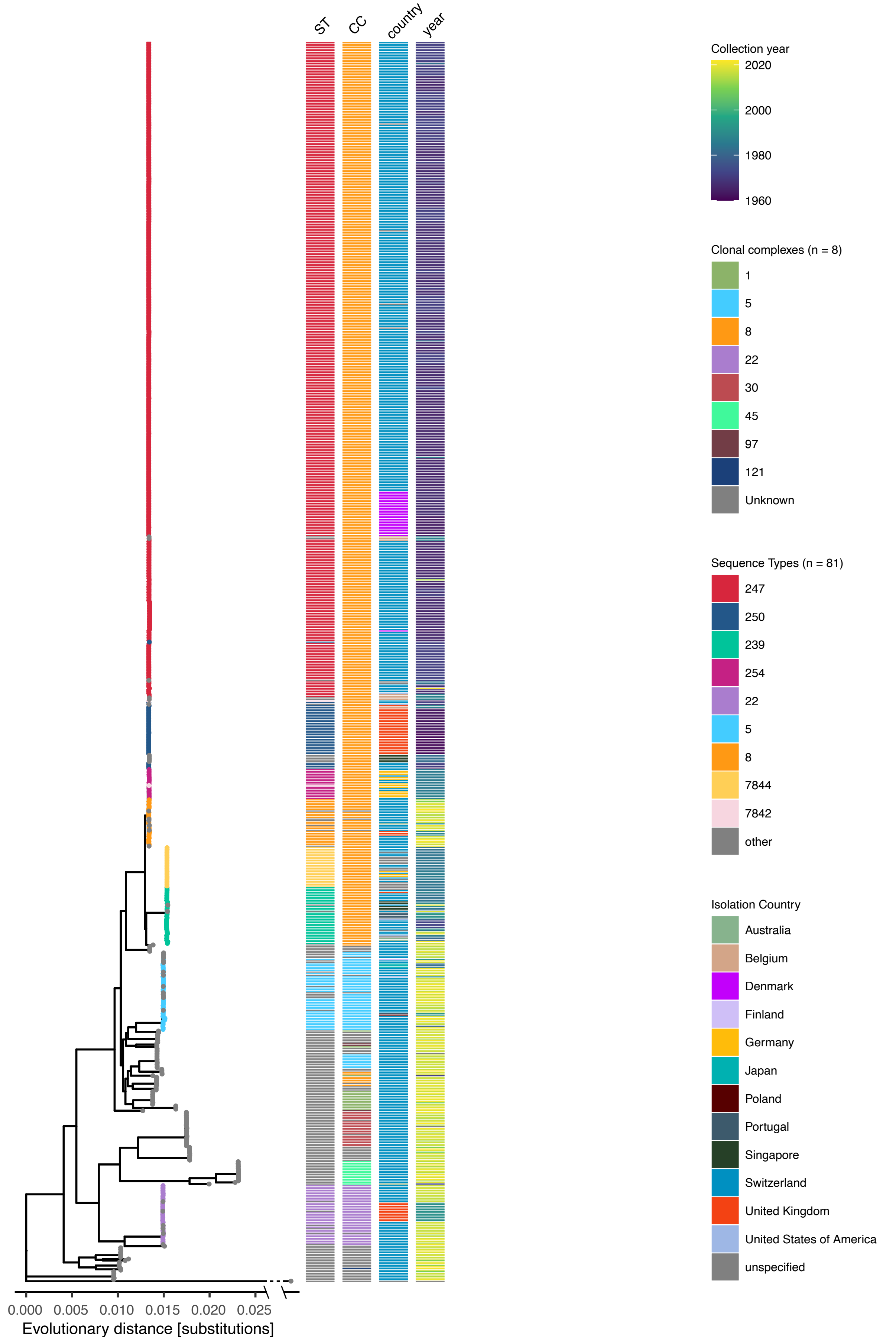

G. MLST sequence type, clonal complex, collection country and collection year mapped to a maximum likelihood core genome SNP tree of MRSA from the CCoS, public repositories and dereplicated modern Swiss isolates (1960 -2022, n = 777). Leaves colored by sequence type. Outgroup (S. epidermis) line shortened through X-axis break for visualization purposes.

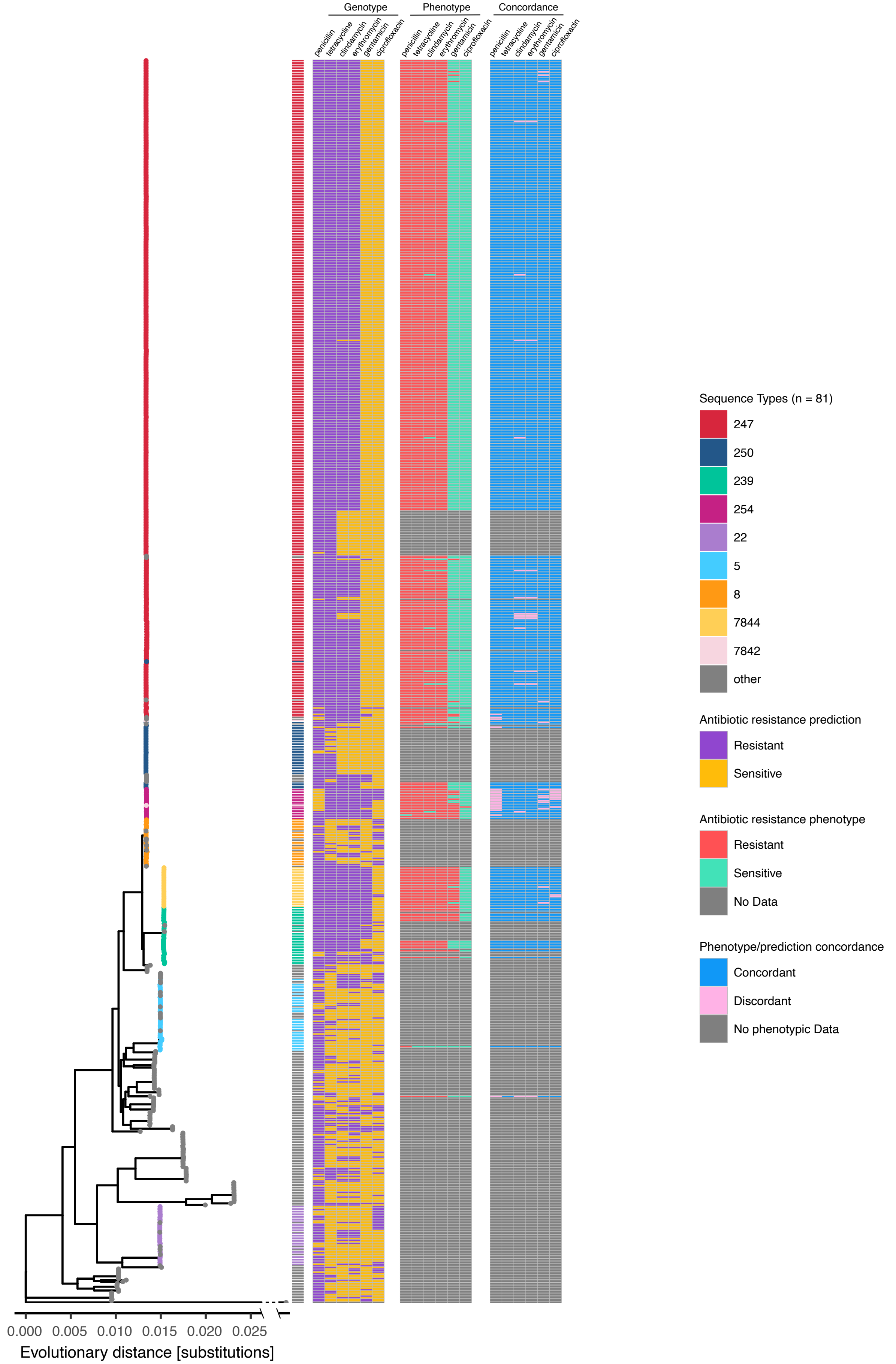

H. Predicted resistance/sensitivity, phenotypical resistance/sensitivity and concordance between the two mapped to a maximum likelihood core genome SNP tree of MRSA from the CCoS, public repositories and dereplicated modern Swiss isolates (1960 -2022, n = 777). Leaves colored by sequence type. Outgroup (S. epidermis) line shortened through X-axis break for visualization purposes.

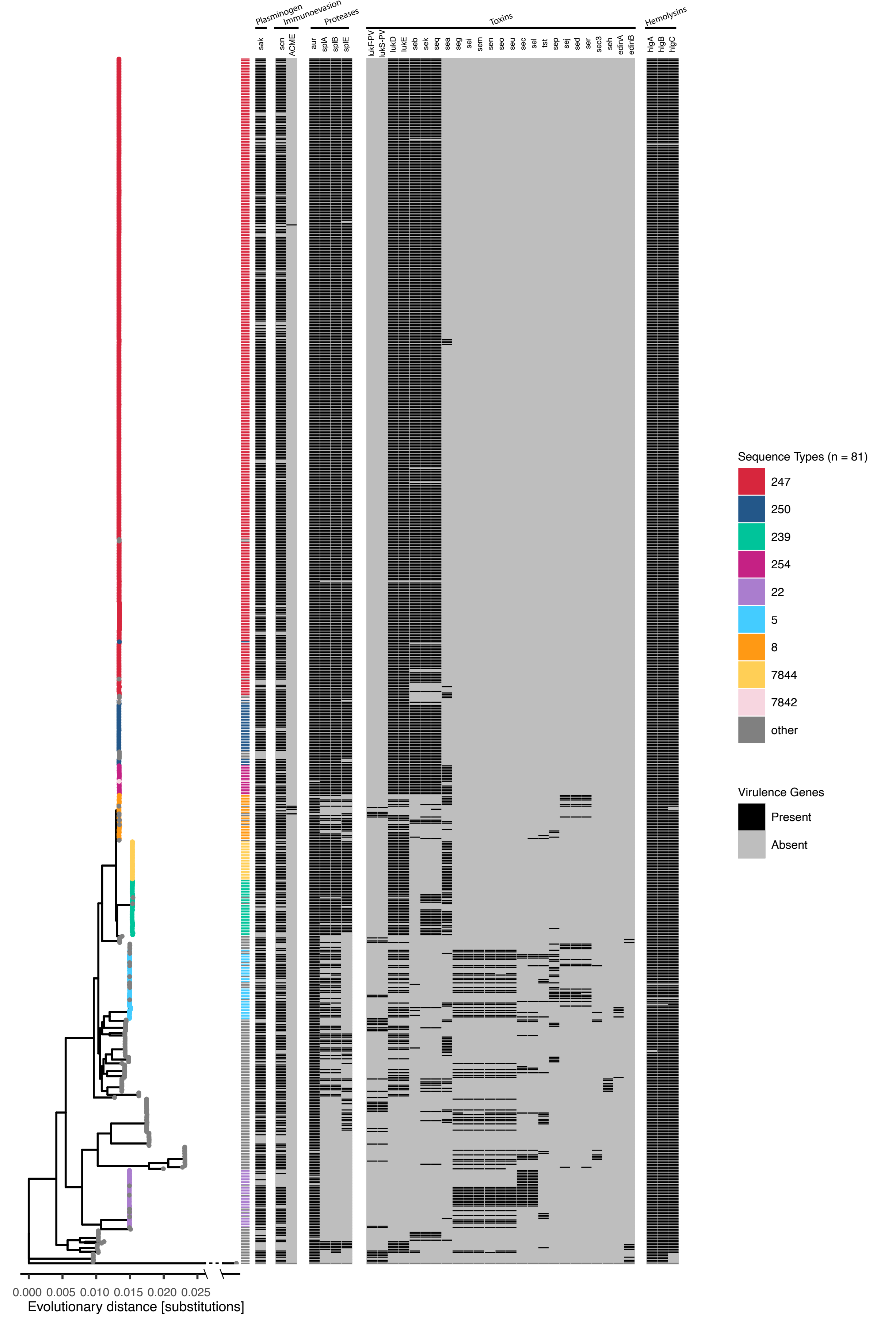

I. Virulence gene presence/absence heatmap mapped to a maximum likelihood core genome SNP tree of MRSA from the CCoS, public repositories and dereplicated modern Swiss isolates (1960 -2022, n = 777). Leaves colored by sequence type. Outgroup (S. epidermis) line shortened through X-axis break for visualization purposes.

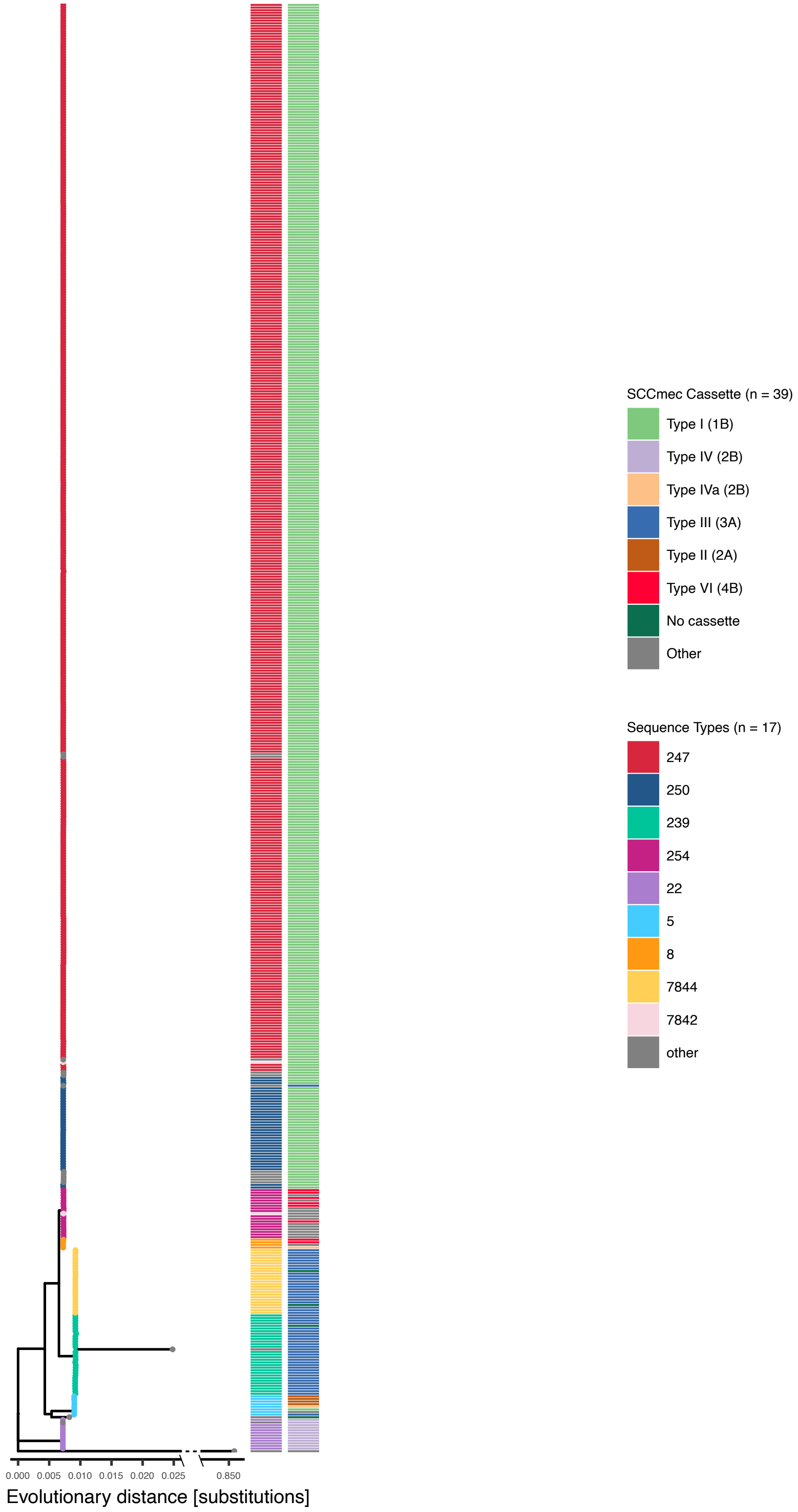

J. SCCmec type mapped to a maximum likelihood core genome SNP tree of ancient Swiss and international MRSA (1960 -1992, n = 554). Leaves colored by sequence type. Outgroup (S. epidermis) line shortened through X-axis break for visualization purposes.

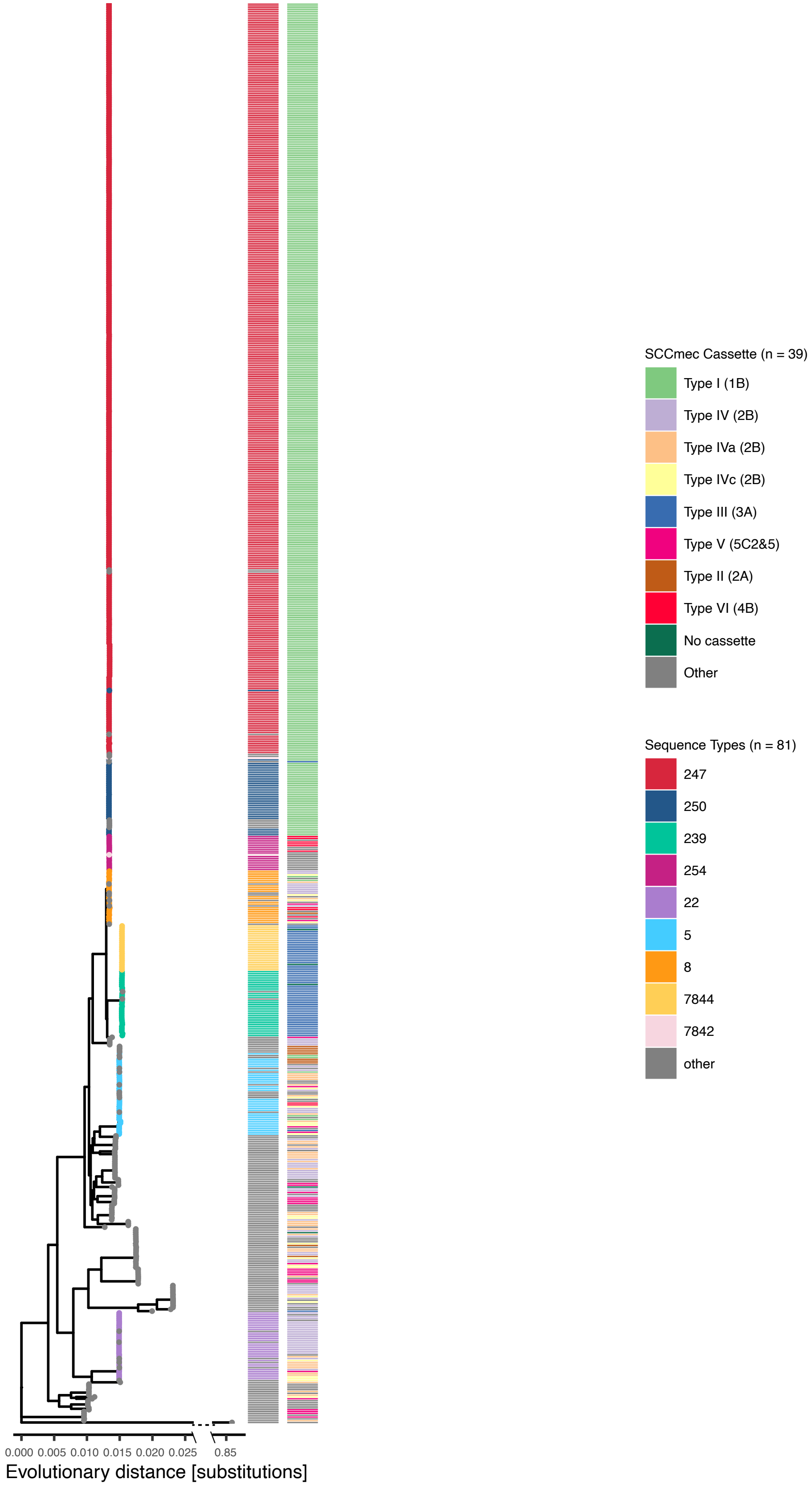

K. SCCmec type mapped to a maximum likelihood core genome SNP tree of MRSA from the CCoS, public repositories and dereplicated modern Swiss isolates (1960 -2022, n = 777). Leaves colored by sequence type. Outgroup (S. epidermis) line shortened through X-axis break for visualization purposes.

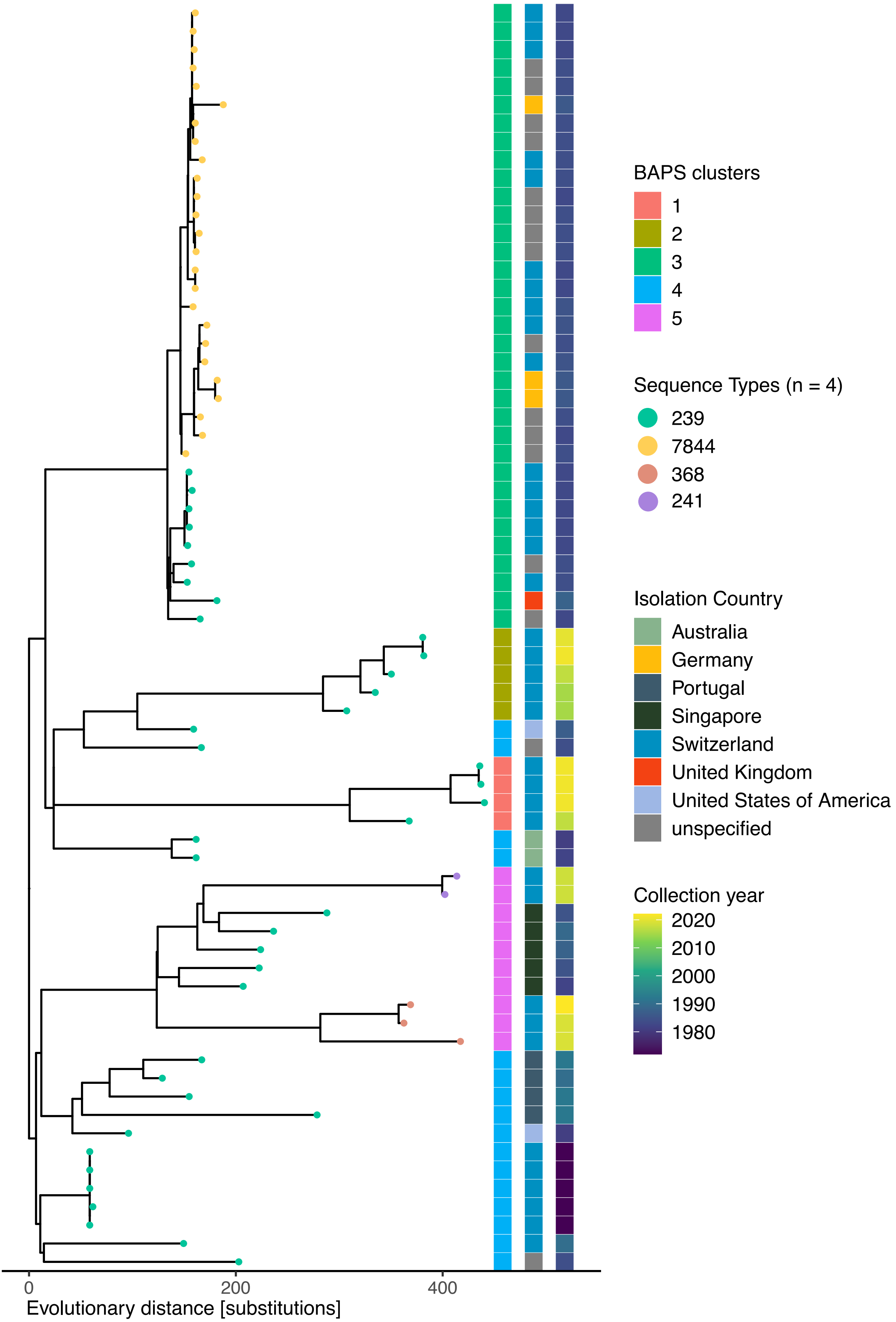

L. BAPS clusters, collection country and collection year mapped to a maximum likelihood whole genome SNP tree of ST239, ST7844, ST368 and ST241 MRSA (n = 69). Leaves colored by sequence type.

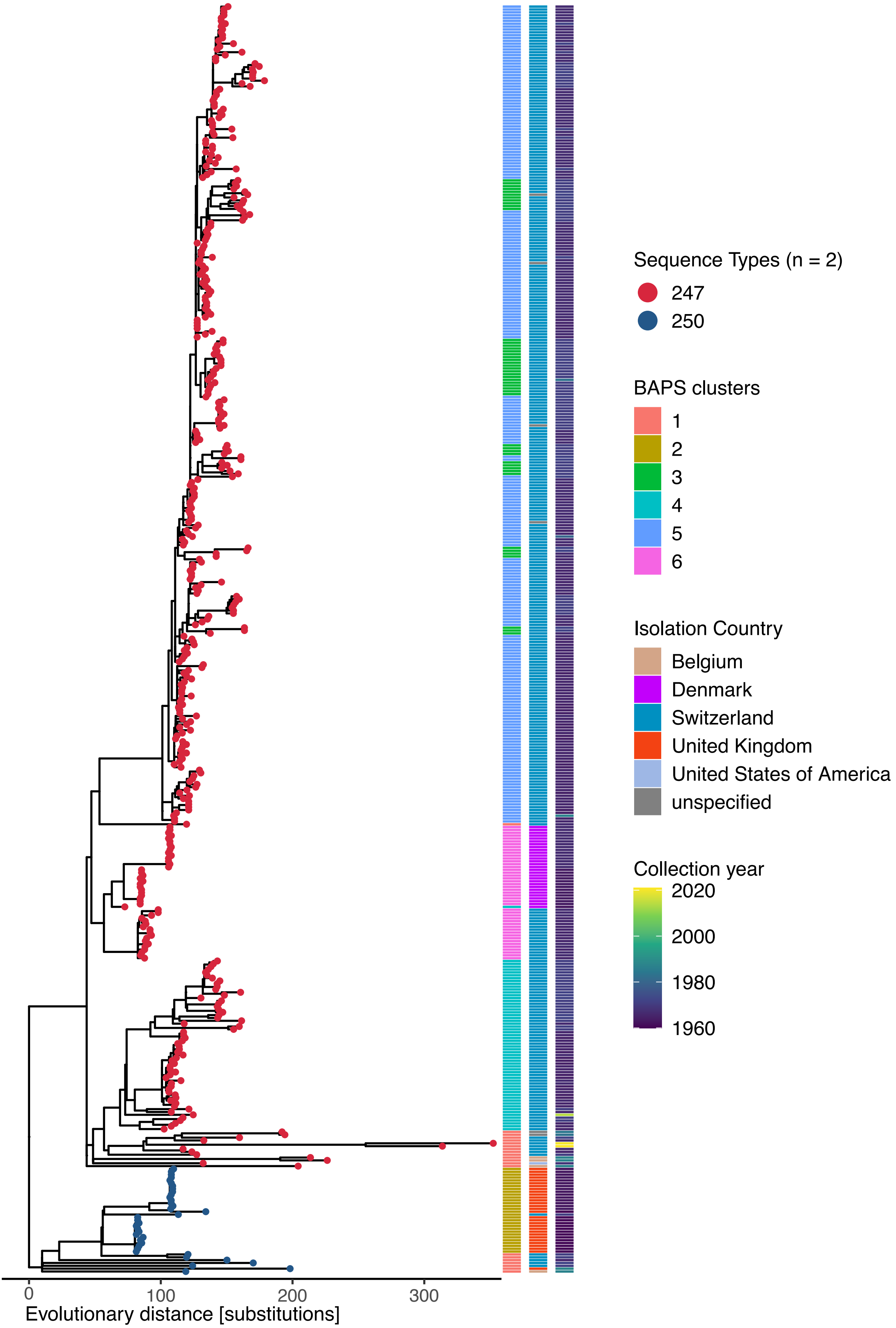

M. BAPS clusters, collection country and collection year mapped to a maximum likelihood whole genome SNP tree of ST247 and ST250 MRSA (n = 445). Leaves colored by sequence type

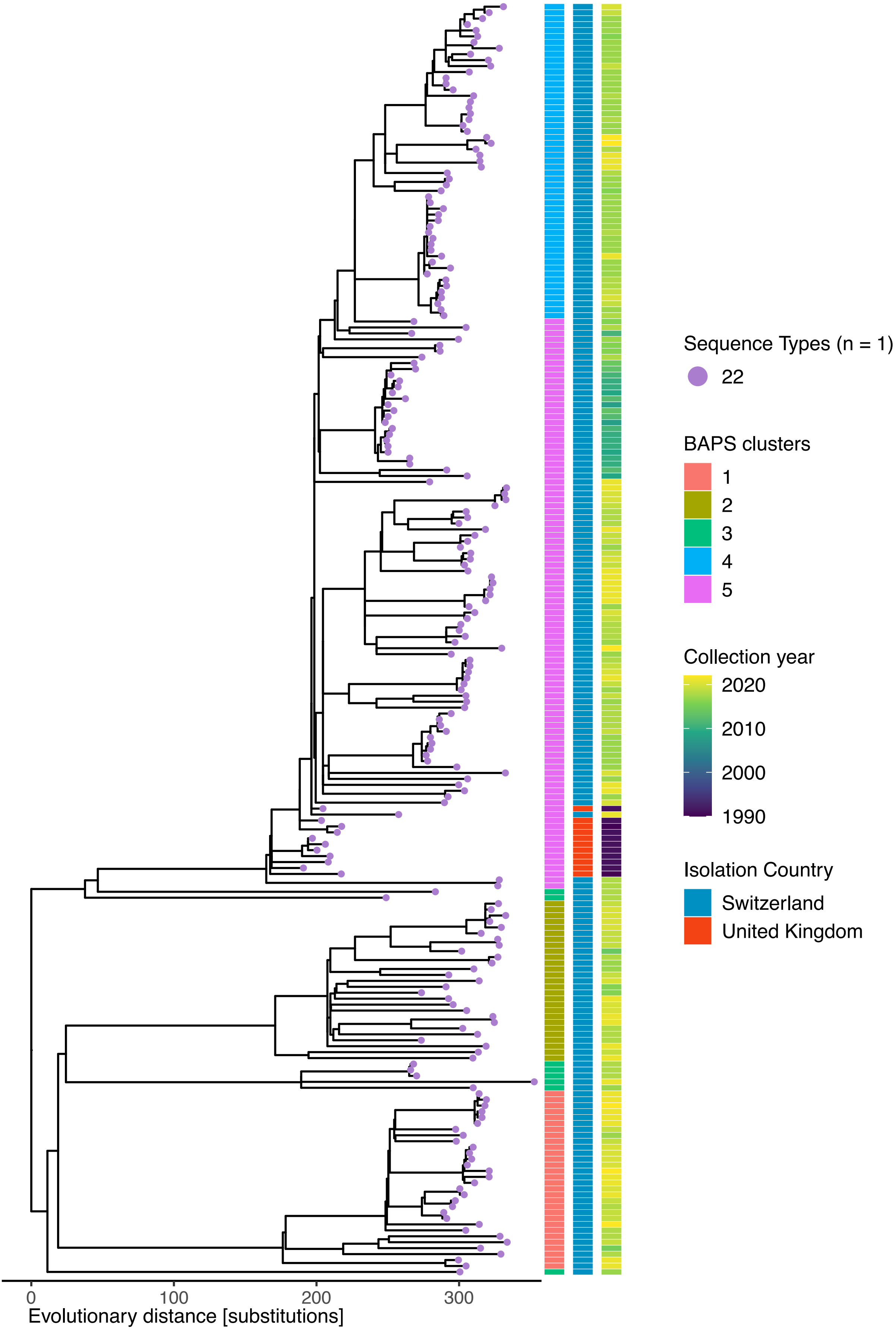

N. BAPS clusters, collection country and collection year mapped to a maximum likelihood whole genome SNP tree of ST22 MRSA (n = 214). Leaves colored by sequence type.
